# Neurodevelopment in Schizophrenia: The Role of the Wnt Pathways

**DOI:** 10.2174/1570159X113119990037

**Published:** 2013-09

**Authors:** Isabella Panaccione, Flavia Napoletano, Alberto Maria Forte, Giorgio D. Kotzalidis, Antonio Del Casale, Chiara Rapinesi, Chiara Brugnoli, Daniele Serata, Federica Caccia, Ilaria Cuomo, Elisa Ambrosi, Alessio Simonetti, Valeria Savoja, Lavinia De Chiara, Emanuela Danese, Giovanni Manfredi, Delfina Janiri, Marta Motolese, Ferdinando Nicoletti, Paolo Girardi, Gabriele Sani

**Affiliations:** 1 NESMOS Department (Neuroscience, Mental Health, and Sensory Organs), Sapienza University, School of Medicine and Psychology, Sant’Andrea Hospital, Rome, Italy;; 2 Centro Lucio Bini, Rome, Italy;; 3 NEUROMED, Pozzilli, Isernia, Italy;; 4 Department of Neuropharmacology, Sapienza University, School of Medicine and Pharmacy, Rome, Italy;; 5 IRCCS Santa Lucia Foundation, Department of Clinical and Behavioural Neurology, Neuropsychiatry Laboratory, Rome, Italy

**Keywords:** Antipsychotic Drugs, Neurodevelopment, Schizophrenia, Wingless (Wnt) signalling.

## Abstract

**Objectives.:**

To review the role of Wnt pathways in the neurodevelopment of schizophrenia.

**Methods:**

Systematic PubMed search, using as keywords all the terms related to the Wnt pathways and crossing them with each of the following areas: normal neurodevelopment and physiology, neurodevelopmental theory of schizophrenia, schizophrenia, and antipsychotic drug action.

**Results:**

Neurodevelopmental, behavioural, genetic, and psychopharmacological data point to the possible involvement of Wnt systems, especially the canonical pathway, in the pathophysiology of schizophrenia and in the mechanism of antipsychotic drug action. The molecules most consistently found to be associated with abnormalities or in antipsychotic drug action are Akt1, glycogen synthase kinase3beta, and beta-catenin. However, the extent to which they contribute to the pathophysiology of schizophrenia or to antipsychotic action remains to be established.

**Conclusions:**

The study of the involvement of Wnt pathway abnormalities in schizophrenia may help in understanding this multifaceted clinical entity; the development of Wnt-related pharmacological targets must await the collection of more data.

## 1. INTRODUCTION 

### 1.1 Neurodevelopment in Schizophrenia

A neurodevelopmental hypothesis for schizophrenia finds its bases in observations and speculations dating back to the mid-sixties [1]. In the early eighties, Irwin Feinberg [2] proposed a defect in synaptic pruning, during a critical period spanning from childhood to adolescence, as the basis of many symptoms of schizophrenia. Further on, Daniel Weinberger [3] in the United States and Robin Murray in the United Kingdom [4] advanced neurodevelopmental hypotheses to explain the coexistence of both positive and negative symptoms in the same patients, symptoms which were previously attributed to antithetic neurochemical mechanisms [5]. These were refined through the years [6-28], while Feinberg’s original hypothesis was revisited and enriched with new ideas [29]. According to the neurodevelopmental hypothesis, schizophrenia represents the outcome of faulty brain development and maturation (and possibly, programming) occurring prior to clinical onset. The multitude of symptoms would arise from an interaction between genetic and environmental factors. The neurodevelopmental model of schizophrenia is supported by several genetic, preclinical, clinical, histological, and neuroimaging data. More precisely, perinatal and obstetric complications [30], prenatal infections [31, 32], cognitive-behavioural and motor signs and symptoms during childhood and adolescence [33], and the persistence of developmental markers beyond the time for which they are expected to be active [34], were reported in many patients with schizophrenia, although their relationship with the development of schizophrenia might be more complex than a simple association (for example, see references [35 and 36]). There is evidence that abnormal brain differentiation and development already occur during pregnancy. In fact, using prenatal ultrasound scans, as well as neonatal structural magnetic resonance imaging (MRI) and diffusion tensor imaging (DTI), differences in cerebrospinal fluid (CSF)-occupied spaces were found between male neonates with and without genetic risk for schizophrenia [37]. However, highly complex biological processes, like neurodevelopment, are unlikely to be driven by one single gene or factor.

### 1.2. The Wnt Signalling Pathway and Development

Although molecules of the Wnt signalling pathway are best known for their roles in embryogenesis and tumori-genesis, they are also involved in several aspects of develop-ment and adult physiology, including cell differentiation and cell polarity generation. The Wnt pathways are conventionally classified as canonical and non-canonical according to whether β-catenin is the principal target of the cascade or not, respectively, but the functional subdivision of Wnt signalling has no basis, as the signalling through the various participating molecules is integrated [38]. The Wnt/β-catenin pathway plays a critical role in the regulation of cell proliferation and in cell fate specification during development, including neurodevelopment [39]. There are many non-canonical, β-catenin-independent pathways, which are activated by Wnt4, Wnt5a, and Wnt11; the best-studied pathways are the Planar Cell Polarity (PCP) and Wnt/Ca^2+^ pathways [40, 41]. In the Wnt/Ca^2+^ pathway, Wnt5a signalling through frizzled (Fzd) and dishevelled (Dvl) regulates intracellular Ca^2+ ^levels determining Ca^2+ ^release from intracellular stores and causing activation of phospholipase C (PLC), leading to the generation of diacylglycerol (DAG) and inositol 1,4,5-trisphosphate (IP_3_), the function of which is to increase intracellular Ca^2+ ^concentration [42]. The non-canonical ligand Wnt5a showed the ability to inhibit the canonical pathway through its binding and internalisation of Ror2, an orphan tyrosine kinase, and subsequent c-Jun-N-terminal kinase (JNK) activation; the whole process ensues in the inhibition of downstream nuclear effects of β-catenin, namely TCF-dependent transcriptional activity and cyclin D1 expression [43]. In the PCP pathway, ligands bind also to Fzd and Dvl to promote scaffold protein-Daam1 complex formation, which activates the G-protein Rho. This protein activates ROCK (Rho-associated kinase), a major cytoskeletal regulator. The most important effects on the cytoskeleton are represented by ROCK binding to actin, which leads to its polymerisation [44-47]. PCP signalling and Wnt/β-catenin signallings are central in the development of the organism. They are strongly associated with body axis elongation and neural tube formation through the process of convergent extension [48, 49]. There are four major classes of secreted extracellular signalling molecules which are expressed in the developing brain during embryogenesis and participate in the patterning of the nervous system, i.e., Wnts, fibroblast growth factors (FGFs), Sonic Hedgehog (SHH), and Bone Morphogenetic Proteins (BMPs) [50]. Wnts, like BMPs, stem from the cortical hem, comprising the medial margin of each hemisphere. Wnt signalling has proven to be essential for neural development at various stages; the first evidence came from a study reporting hypotrophy of the cerebellum and midbrain in knockout-for-the-*Wnt–1*-gene mice [51]. Subsequently, a general principle of Wnt signalling during brain development has been discovered, namely, that regional specification needs an anteroposterior gradient of Wnt signalling, i.e., low for the anterior structures and high for the posterior ones [52, 53]. Wnt molecules are involved not only in patterning, but also in morphogenesis, neuronal migration and specification [54-56], neuronal precursor proliferation [55], synaptic differentiation [57, 58], and mature synapse modulation [59-61]. Their proliferation-promoting role is central in stem cell maintenance and progenitor pool expansion. Furthermore, Wnt signalling is involved in differentiation processes and lineage decision events during both central and peripheral nervous system development. In the adult brain, it may select neural circuitry formation through the regulation of long-term potentiation [62-64]. This matches its action in axon guidance, neurite outgrowth, and synaptic plasticity (recently reviewed in ref. [65]). The Wnt signalling pathway is also essential in maintaining proper communication between neuronal and non-neuronal cells; a distruption of this balance in advanced age may lead to neurodegeneration and related disorders, such as Alzheimer’s disease [66, 67].

The purpose of our review was to identify possible connections between schizophrenia and molecules related to the Wnt pathway in order to establish the hierarchy of this developmental pathway in the pathophysiology of this complex disorder.

## 2. METHODS

To identify studies of Wnt-related molecules, we searched the PubMed database using all Wnt-related terms and crossing them with each of the specific targets of our review, i.e., all antipsychotics and all disorders of the schizophrenic spectrum. Identified papers’ reference lists were searched subsequently to identify further relevant literature.

We proceeded similarly for other sections of this review. To review the role of the Wnt pathway in human physiology and normal development, we crossed the above Wnt-related set with other key words, like neurodevelopment, development, apoptosis, cellular death, proliferation, oncogenesis, physiology, nervous system, embryogenesis etc. To review the neurodevelopmental theory of schizophrenia we crossed the key word schizophrenia with terms like neurodevelop-ment, neurodevelopmental, development, developmental, CNS, and neurodegeneration. There were no timeframe limitations in our searches.

## 3. ROLE OF INDIVIDUAL WNT SIGNALLING MOLECULES IN DEVELOPMENT AND NEURO-DEVELOPMENT

### 3.1. Wnt1: Wnt1 is a Secreted Glycoprotein and a Member of the Wnt Protein Family 

In the human, it is encoded by the evolutionally highly conserved *WNT1* gene. Wnt1 acts through the Frizzled (Fdz) family of receptors. Originally, Wnt1 has been identified as a proto-oncogene whose overexpression contributed to mammary oncogenesis [68]. Its interaction with Wnt3a is suggested to be important for neural tube patterning [69]. *In vivo* data from transgenic mouse lines and explant cultures show that Wnt1 is required for establishment and differentiation of midbrain dopaminergic neurons during embryogenesis [70].

### 3.2. Wnt2: Wnt2 is a Member of the Wnt Family

The human gene encoding Wnt-2 has been identified through its sequence similarity with the murine protoonco-gene *Wnt1* gene and its Drosophila ‘wingless’ homologue [71]. However, its role in schizophrenia is unclear, since its polymorphisms were not found to be associated with this disorder [72].

### 3.3. Frizzled Family

The Frizzled gene family, mostly through the mediation of Wnt signaling, is involved in the development of various structures. There are several kinds of Wnt receptors, principally belonging to the Frizzled (FZD) family, that regulate both the canonical and non-canonical pathways [73, 74]. The Frizzled family receptors comprise ten different receptors that mainly modulate the canonical pathway [75]. The *FZD3* gene consists of eight exons and is approximately 70 kb long; it encodes for the FZD3 receptor, one of the major components of the Wnt signaling pathway, which is involved in the regulation of early neurodevelopmental processes. FZD3 is an almost ubiquitous transmembrane receptor for Wnt ligands implicated in the specification of several structures, like neural crest derivatives, and in the correct axonal targeting during the development of major forebrain fibre tracts [76-80]. The *FZD3* gene spans approximately 60 kb of DNA and about 40 SNPs are registered in the SNP database of the US National Center for Biotechnology Information.

Frizzled proteins possess an outer N-terminus and seven transmembrane domains, concluding with a C-terminal intracytoplasmic tail involved in cellular signalling [81]. Only for a few of these multiple FZDs has their role in development been elucidated, primarily in invertebrates. In the nematode, *Caenorhabditis elegans*, FZDs regulate cell fate, asymmetric cell divisions, and spindle pole orientation [82]. Much less is known about their roles in vertebrate development. In the highly aquatic amphibian, *Xenopus*, Xfz-10 promotes sensory neuron development [83]; maternal Xfz7 is required for dorsal axis development and for response to ectopic Xwnt8b [84], while Xfz8, which is expressed in the Spemann organizer, appears to organise cell movements during gastrulation [85], in dorsal development [86]. It is also required for oligodendrocyte development in the vertebrate zebrafish spinal cord [87]. Wnt/Frizzled signalling is involved through Xfz3 in early entraining neural crest development and specification [77-79]. Xfz3-interacting protein Kermit [88] also shows that Xfz3 signaling is required for neural crest formation. Last, Xfz3, mediates eye development in the *Xenopus* [89]. In the *Cyprinida* zebrafish, FzA, which is closely related to Xfz8, Xfz3 may play a role in dorsoventral patterning [90]; Fz7a shows both maternal and zygotic strong expression within the anterior neuroectoderm and is more weakly expressed within the lateral mesoderm. During somitogenesis, it is detected in the developing central nervous system (CNS), including the somatic and posterior lateral mesoderm, and the migrating lateral line primordium [91, 92]. Finally, F8 is required for oligodendrocyte development in the zebrafish spinal cord [87].

However, it is unclear which endogenous ligands activate these or any other vertebrate FZDs. Furthermore, ligands related to canonical and non-canonical pathways may compete for FZDs and exert opposite effects to Wnt-mediated intracellular translation [93], thus forming a complex network of Wnt molecules and FZDs to control different actions [94] and novel ligands are continuously discovered to render the whole picture extremely complex [95].

In mammals, two of the most complex roles are the specification of neurons within the CNS and the correct targeting of their axons. Despite the large number of FZDs and their widespread expression within the CNS [96-98], only a few reports investigated their role in specification and axon targeting. Fz3 knock-out mice express massive defects in the development of fibre tracts within the forebrain, while leaving other aspects of CNS development mostly unaffected [80].

### 3.4. Low-density Lipoprotein Receptor 5/6 (LRP5/6)

The low-density lipoprotein (LDL) receptor is a member of a family composed of seven structurally closely related transmembrane proteins (LRP1, LRP1b, megalin/LRP2, LDL receptor, very low-density lipoprotein receptor, MEGF7/LRP4, and LRP8/apolipoprotein E receptor2). Wnt proteins bind Frizzled (FZD) and LDL-receptor related protein (LRP)5/6 cell surface receptors and prevent glycogen synthase kinase (GSK)-dependent phosphorylation of β-catenin, thus leading to its stabilisation [99]. Regarding neurodevelopment, LRPs may be involved in signal transduction during early neuronal differentiation [100]. In *Xenopus* embryos LRP6 activated Wnt-Fz signalling and induced Wnt responsive genes, dorsal axis duplication and neural crest formation. In addition, a LRP6 mutant lacking the carboxyl intracellular domain blocked signalling through Wnt or Wnt-Fz, but not through Dishevelled or β-catenin, thus inhibiting neural crest development [101]. Anti-LRP antibody inhibited neurite outgrowth of primary hippocampal neurons, cultured in either serum-containing medium or in cortical astrocyte monolayers in serum-free medium [102].

### 3.5. Dishevelled-1 (DVL1)

Signalling through Frizzled and Ryk requires the cytoplasmic scaffold protein Dishevelled [103]. However, the discovery of new Wnt receptors [104] casts doubts on whether Dvl is an absolute requirement for all Wnt-mediated functions. In the canonical pathway, Wnt molecules activate Dvl to inhibit the serine/threonine kinase GSK3β, with subsequent cytoplasmic elevation and translocation of β-catenin to the nucleus, to regulate transcription [105]. Dvl is localised in various cellular compartments, like the cytosol, the nucleus, and the presynapse [106-108] and can bind to microtubules to increase their stability in both dividing cells and differentiating neurons [109]. In addition, Dvl regulates dendritic morphology in hippocampal neurons [110]. Loss of function of *Dvl1*, one of the three mouse *Dvl* genes, results in social interaction deficits [111].

### 3.6. Glycogen Synthase Kinase-3 (GSK3)

Glycogen synthase kinase-3 (GSK3) proteins are serine/threonine kinases that were originally identified as key regulatory enzymes in glucose metabolism [112, 113]. In rodents and humans, an alternative splice variant of GSK3β, GSK3β2, is specifically expressed in the nervous system; its highest levels are found during development [114]. Recent studies suggest that GSK3β2 plays a specific part in neuronal morphogenesis *in vitro* [115, 116]. GSK3 proteins and their upstream and downstream regulators play key roles during neurodevelopment and neuroplasticity, including neuro-genesis, neuronal migration, neuronal polarisation and axon growth and guidance. Disruption of GSK3 signalling adversely affects brain development and is associated with several neurodevelopmental disorders [117]. GSK3 is at the crossroads of many a pathway of intracellular signalling, including Wnt–β-catenin, Sonic Hedgehog, Notch, PI_3_K, ρ-GTPases, mTOR, Akt, PKA-LKB1, thus affecting intracellular events in a very complex way. As a result, it plays multiple roles in both neurodevelopment and morphogenesis, in form maintenance and remoulding, in homoeostatic function during adult life [118, 119], and also in response to various treatments, including mood stabilising and antipsychotic drugs [120, 121].

### 3.7. Adenomatous Polyposis Coli (APC)

*APC* was originally identified as a tumour suppressor gene mutated in familial adenomatous polyposis, an autosomal dominant condition with predisposition to colorectal cancers [122] and brain tumours [123]. It is a key component of the APC/AXIN/CKI/GSK3β destruction complex and a negative regulator of the Wnt signalling. APC protein facilitates growth and proliferation of neurons *in vitro*. Mutations in APC have also been linked to a case of mental retardation [124]. Many genes whose dysfunction is associated with tumour formation have important functions in the normal development of the nervous system [125]. Apc (the murine homologue of *APC*) is known to be involved in regulating a variety of cellular processes, including mitosis, cytoskeletal dynamics, axonogenesis, cell polarity, and apoptosis [126, 127]. Given the multiplicity of Apc functions, it affects the development of cerebral cortical cells in a multitude of ways. Apc is central to the Wnt signalling pathway, where it mediates the breakdown of cytoplasmic β-catenin protein [105], thus countering Wnt signals which stabilise β-catenin and allowing it to translocate to the nucleus and activate the transcription of target genes in collaboration with TCF/LEF. With cerebral cortical Apc depletion, important changes in cell polarity and cytoskeletal organisation occur in combination with changes in β-catenin activity; these result in reduced cortical size, disorganised cell connectivity, shift in cell identity (neurons lose their prefrontal characteristics to assume those of posterior brain) [128]. Interestingly, Apc heterozygous knockout mice display working memory deficits (a condition shared by people with schizophrenia) when they are 11-12 weeks-old, an age roughly corresponding to 16-18 years of age in humans, but not at 6-7 weeks of age, which is about 10 years for humans [129]; the parallel with age of onset of schizophrenia is impressive.

### 3.8. β-Catenin

β-catenin is a protein that in humans is encoded by the *CTNNB1* gene. The nomenclature of the homologous protein in Drosophila is armadillo. β-Catenin is a subunit of the cadherine protein complex and is an integral component in the Wnt signaling pathway [130]. Tyrosine-142 phosphorylation of β-catenin is the initial event promoting the release of this protein from the cell membrane, thus rendering it available as a transcription factor [131]. The enzyme which reverses this step is the tyrosine phosphatase, RPTP β/ζ [132]. Pleiotrophin is a natural inhibitory ligand for RPTP β/ζ [132]. Pleiotrophin thus acts as a growth factor by enabling β-catenin release from the plasma membrane. β-Catenin is involved in developmental processes such as embryonic pattern formation, determination of cell fate, and articular cartilage function [133-135].

### 3.9. Transcription Factor-4 (TCF4)

Transcription factor-4 (TCF-4) is a protein member of the basic helix-loop-helix (bHLH) transcription factor family, binding DNA at CANNTG motifs, also termed E-Boxes [136]. TCF-4 is broadly expressed during early human development throughout the CNS, the sclerotome, peribronchial and kidney mesenchyme, and the genital bud [137]. TCF-4 isoforms control the development of different parts of the brain, i.e., midbrain and isthmus in the developing *Xenopus*; in this context, canonical Wnt signalling autoregulation and alternative expression of different isoforms of XTcf-4 are essential for developing CNS specification [138].

### 3.10. Dickkopf-1 (DKK1)

The secreted protein Dickkopf1 (Dkk1), a member of a multigene family, which induces head formation in amphibian embryos [139], was first found to inhibit Wnt in the late nineties [140]. Later, its inhibition of Wnt/β-catenin signalling was found to occur through binding to the LRP6/arrow Frizzled coreceptor moiety [141]. DKK1, together with Wnt/β-catenin signalling, plays an important role in antero-posterior (a-p) patterning of the primary embryonic axis. The different expression of Wnt pathway molecules and their antagonists, such as DKK1, create signalling gradients, which induce correct axial structure formation, in particular, head development. In the mouse embryo, DKK1 is expressed from the gastrula to the neurula in the anterior visceral endoderm, the anterior mesendoderm, and foregut endoderm. The expression of DKK1 in the anterior mesendoderm of the mouse is required for early anterior development, but is not essential in the anterior visceral endoderm for proper anterior morphogenesis [139]. Canonical Wnt signals and DKK1 function as repulsive and attractive guidance cues, respectively, during visceral endoderm cell migration. This supports a model where a signalling gradient of Wnt/β-catenin and DKK1 mediates a-p axis polarization by guiding anterior cell migration in the pregastrula mouse embryo.

### 3.11. Dikkopf-3 (DKK3)

Despite genetic and structural similarities between Dikkopf-3 (DKK3) and other Dikkopfs, its relation to Wnt signalling is unclear [142]. *In situ* hybridisation showed significant DKK3 expression in mouse cortex, hippocampus, and brain stem [143]. The role of DKK3 in the nervous system is far from clear. Interestingly, elevated plasma and cerebrospinal fluid DKK3 levels have been observed in Alzheimer’s disease [144].

### 3.12. Dickkopf-4 (Dkk4)

Dickkopf-4 (DKK4), like other members of this family, is a negative regulator of Wnt signaling. It appears to act like Dkk1, moderating Wnt signaling in the developing forebrain [145]. Its role during embryogenesis, despite specifically-focused studies, remains obscure. DKK4 acts in a negative feedback loop to attenuate canonical Wnt signalling, and may facilitate switch to the non-canonical Wnt planar cell polarity (PCP) pathway, which is involved in cell movements during morphogenesis [146]. 

### 3.13. Secreted Frizzled Related Protein-1 (sFRP1)

The sFRPs belong to the largest family of Wnt inhibitors. sFRP-1, an antagonist of Wnt signalling which is under control of the Hedgehog pathway [147] and inhibits β-catenin accumulation through a GSK-3-dependent mechanism [148], binds directly to Wnt molecules to interfere with its binding to Frizzled receptors [149]. It regulates cell proliferation, differentiation and apoptosis, but its deletion was not shown to have any obvious effect on the gross anatomy of the nervous system in mice [150]. sFRP-1 mRNA expression in the developing mouse neocortex occurs during the entire developmental period and it is spatially restricted to the proliferative zones [151]. Furthermore, sFRP-1 and sFRP-3 are expressed in anterolateral to caudomedial gradients within the telencephalic ventricular zone during corticogenesis [152]. The human chromosomal region 8p, which is involved in the genetics of neuropsychiatric and other diseases [153, 154], contains at least three Wnt signalling-related genes (*FZD3*, *DKK4*, and *SFRP1*). Summarising, sFRP-1 influences many processes around the body, but is essential for none. This could be related to functional redundancy and to the vicarious functions of other similar proteins involving sFRPs and Dkks [155-156]. Furthermore, it appears to have functions related to both growth and breakdown, thus contributing to morphogenesis and remoulding, but its effects are temporally limited and developmental state-related [157], and in a later stage its actions may oppose its earlier ones [158].

Wnt signaling appears to be very important during neurodevelopment, but it also affects synaptic plasticity and neurogenesis during adult life and controls excitatory synaptic transmission in a key area, like the hippocampus [61, 159, 160], which is believed to be involved in the pathophysiology of schizophrenia [161, 162]. Presynaptic effects of canonical Wnt signaling and postsynaptic Wnt non-canonical signalling concur in excitatory neuro-transmitter release in adult hippocampus and in postsynaptic density 95 (PSD-95)-glutamate receptor incorporation [67] and both these mechanisms contribute to maintenance of long-term potentiation, the first through mTOR activation secondary to GSK3 suppression [163], the second with a Ca^2+^-dependent non-canonical Wnt activation [63]. It should be recalled that schizophrenia is accompanied by long-term potentiation deficit [164]. Hence, it is possible that deficits which first appeared early in development and which depended on faulty molecular communication on a possible genetic basis, continue to be involved in maladaptive behaviour in adult life. There is evidence from a ground-breaking paper that such defects regard the genoma of people with psychotic disorders, as they are present even in fibroblasts reprogrammed to become neurons derived from such patients [165]. Interestingly, in this paper, altered gene expression was found in both NMDA receptor genes and in several genes encoding Wnt-related molecules and other molecules which are closely related to pathways interacting with Wnt signaling. These exciting discoveries pave the way for the identification of genetic markers of abnormal behaviour and may provide a clue in the future as to the nature of what we currently call schizophrenia.

## 4. THE WNT SIGNALLING PATHWAY AND SCHIZOPHRENIA

An alteration in one or more of Wnt pathway genes may affect brain development and be related to several neurodevelopmental disorders [117]. In particular, the role of the Wnt pathway in the regulation of neuronal migration during development suggests its possible involvement in the cytoarchitectural defects observed in schizophrenia [166]. In fact, the Wnt pathway (and GSK3 in particular) is also involved in programmed cell death, an essential component of normal brain development. The abnormal brain neuronal distribution reported from post-mortem samples of patients with schizophrenia may result from aberrant programmed cell death [167]. Preventing apoptosis through GSK3 inhibition has been advocated as a possible therapeutic target to protect against psychiatric disorder-related neuro-pathology [168]. GSK3β is involved in serotonin deficiency-related aberrant behaviour, since its inhibition alleviates such behaviour [169], while it plays a part in dopamine-mediated hyperlocomotion, an effect which is blocked by anti-psychotic treatment [170]; the interplay between these neuro-transmitters could prove to be crucial in schizophrenia [171, 172], and many psychiatric treatments active in schizophrenia target both monoamine neurotransmission and GSK3 [173]. Lithium and valproate directly inhibit GSK3, whereas clozapine, haloperidol, risperidone, fluoxetine and imipramine increase its inhibitory phosphorylation. Atypical and typical antipsychotic drugs alter GSK3β activity, as do psychosis-inducing drugs; some psychoactive substances decrease GSK3 activity while chronic antipsychotic treatment increases GSK3 activity [174, 175]. The inhibition of GSK3 could represent a key factor in the behavioural and neurotrophic effects of psychotropic drugs and a possible target for treatment [176, 177].

### 4.1. Wnt1

Mutations in Wnt cause neurodevelopmental alterations in mice. Late-gestation foetuses homozygous for mutated *Wnt-1* alleles show a virtual absence of midbrain and cerebellum and newborns die within 24 hours [39]. The only early immunohistochemical post-mortem study performed to date, showed increased Wnt-1 immunoreactivity in hippocampus, in particular in the CA3 and CA4 subregions, in patients with schizophrenia *versus* controls with no clinical or morphologic evidence of brain pathology [34].

### 4.2. Wnt2

The *WNT2* gene is located at chromosome 7q31.2; the 7q31 region showed in the Genome Scan of European-American Schizophrenia Pedigrees a significant linkage to schizophrenia [178], but this is not a sufficiently strong point for the involvement of Wnt2 in schizophrenia. Six studies to date investigated whether the *WNT2* gene is associated with autism, another neurodevelopmental disorder [179, 181-184], with three studies finding rare mutations in the *WNT2* gene to increase significantly the susceptibility to autism [179, 182, 183], while another found a possible interaction between two SNPs, one for *WNT2* and one for *EN2*, in influencing language development in autism [184]. Given the similarities between autistic disorders and schizophrenia [as a neurodevelopmental disorder], H.J. Kim *et al*. [72] investigated whether *WNT2* gene variations are risk factors for schizophrenia in a Korean sample; however, their results suggested that Wnt2 may not be involved in the pathogenesis of schizophrenia. Of all the SNPs and haplotypes analysed, only one SNP, rs4730775, showed a weak association with the disorder [72]. Overall, these results give little support to the existence of a genetic association between Wnt2 and schizophrenia.

### 4.3. Frizzled Receptor

Studies in the last decade focused on the association between SNPs of the human Frizzled 3 gene (*FZD3*) and schizophrenia. The first studies analysed eight SNPs in candidate gene association studies, finding significant associations for SNP rs2323019 (SspI site), rs960914, and rs880481 in Chinese Han populations through a transmission disequilibrium and *chi*-square test [185, 186], while neither allele-wise nor genotype-wise analysis revealed any evidence of association in SNP rs181277, rs352222, rs352226, rs2874940, rs3757884, and rs3757888 [185-188]. A Japanese study found an association between schizophrenia and IVS3+258T>C polymorphism (rs960914) or IVS3+258T−435G haplotype [187]. However, a study in Caucasians, which investigated the SNP rs960914, did not confirm the FZD3 as a risk locus for schizophrenia [188]. Regarding SNPs rs352203 (NlaIII site) and rs2241802 (AluI site), they were first significantly associated with schizophrenia in a Chinese Han population [185], but this was not confirmed in Japanese, British, South Korean and other Chinese populations [186-192]. The frequencies of the rs2241802 SNP allele or genotype were found to differ between healthy controls and patients with schizophrenia in a Va Chinese population [193]. Finally, a recent study analysed the rs7836920 SNP in a Brazilian sample, but found no significant association with schizophrenia [194].

*FZD3* gene SNPs have been also studied in the context of the methamphetamine psychosis model, which shows significant association between the *FDZ3* gene and vulnerability to schizophrenia; two haplotypes comprising SNP rs2241802, rs2323019, rs352203, and rs880481 have been identified as potential risk factors [195], suggesting a role for FZD3 in both schizophrenia and drug-induced psychosis.

### 4.4. Dishevelled1

Dishevelled1 (DVL1), the human homologue of the Drosophila *dishevelled* gene (*Dsh*), encodes a cytoplasmic phosphoprotein, Dvl-1, which is integrated with the Wnt pathway, regulating cell proliferation, transducing developmental processes like segmentation and neuroblast specification [196], cell fate decisions, cell polarity, and microtubular stabilisation [109]. A gene-targeting study of *Dvl*1, showed this mouse gene to be a genetic factor influencing social behaviour and sensorimotor gating in mice; *Dvl*1-deficient mice are viable, fertile, and structurally normal but they exhibit reduced social interaction, including differences in whisker trimming, deficits in nest-building, less huddling contact during home cage sleeping, and subordinate responses in a social dominance test [111, 197]. Sensorimotor gating is abnormal, as measured by deficits in prepulse inhibition of acoustic and tactile startle. Thus, *Dvl*1 mutant mice may provide a model for aspects of several human psychiatric disorders [111]. In humans, hemizygous deletion of a 1.5-3 Mb region of chromosome 22q11.2 may lead to DiGeorge syndrome, which is characterised by learning disabilities, craniofacial abnormalities and congenital heart defects, and who may develop schizophrenia in about one-fourth of cases in adult life [198]. However, caution is needed, because none of the known *DVL* structural genes map to human chromosome 22 or to syntenic mouse regions, and not even to any of the loci presently known to map schizophrenia [111].

Sensorimotor-gating dysfunction (impaired startling response or abnormality in pre-pulse inhibition) is characteristic of schizophrenia, among other psychiatric disorders [199, 200]. Impaired pre-pulse inhibition patients with schizophrenia appears to be a stable trait, independent from treatment and course of the disease [201]. Social impairment is present in schizophrenia and may relate to autistic traits and structural brain abnormalities [202]. It is tempting to speculate that *DVL1* gene abnormalities may underlie some of the complex behavioural abnormalities encountered in schizophrenia, such as impaired response to startling and social interaction deficit, but this is currently unlikely to apply to all patients with schizophrenia.

### 4.5. Akt

Emamian *et al*. [203] showed a reduction in AKT1 protein levels and phosphorylation of GSK3β in Ser9 in post-mortem frontal cortex and hippocampus of patients with schizophrenia; similar changes were observed also in peripheral lymphocytes of patients with schizophrenia, compared to controls. The same study also showed that treatment with haloperidol in mice is associated with increased total Akt phosphorylation. Further studies showed decreased Akt expression in the dorsolateral prefrontal cortex (Brodmann’s area 46) of patients with schizophrenia [204, 205]. Van Beveren *et al*. [206] not only found decreased expression of *AKT1, 2, *and* 3* genes in periferal momonucleate blood cells (PMBCs) of recent-onset (<5 years), male patients with schizophrenia, but also differences between patients and healthy controls in other genes involved in several cellular processes. A total of 1224 genes were found to be dysregulated in patients, with most transcripts down-regulated (*N*=952), and involving many pathways, like JAK/Stat, ERK/MAPK, Wnt/β-Catenin, neuregulin, and VEGF signalling. Interestingly, Akt is involved in many of these processes.

Lower phosphorylated Akt levels were also associated to a decrease in hippocampal volume [207], one of the most frequent structural brain abnormalities in schizophrenia [208, 209]. Another study [210] showed a decrease in phosphorylated Akt in the dentate gyrus, the neurogenetic zone of the hippocampus, in tissue samples from patients with schizophrenia. Structural abnormalities in the prefrontal cortex (PFC) such as impaired dendritic architecture of pyramidal neurons, alterations in the expression of *PFC* genes involved in neuronal development, altered synaptic transmission, abnormal myelination, and deficit in working memory retention under neurochemical challenge of three distinct neurotransmitter systems were reported in Akt1-deficient mice [211].

Despite the apparent importance of Akt in brain physiology and its abnormally low activity in schizophrenia, haloperidol, which is a classical neuroleptic drug, enhances the nuclear translocation of phosphatidylinositol 3'-kinase, which in turn disrupts Akt phosphorylation [212]. It is possible that this action mediates some side effects of this drug, because haloperidol, but not risperidone, was able to increase apoptosis of primary cultured rat cortical neurons through reduction of Akt activity [213].

Genetic linkage analyses showed co-segregation of *Akt1* haplotypes with schizophrenia, suggesting that the *AKT1* gene may be a schizophrenia susceptibility gene [203]. Other genetic studies demonstrated association of *AKT1* gene polymorphisms with schizophrenia in Chinese [214], Irish [205], British [215], Japanese [206], Iranian [217], and European [218] populations. Pinheiro *et al*. [219] examined the potential association of genetic variation in the *AKT1* gene with neurocognition in schizophrenia. Using Emamian’s *et al*. [203] five schizophrenia-associated SNPs in the *AKT1* gene (Table **1**), they found no significant association with multiple neurocognitive domains. More recently, the relationship between these five *AKT1* SNPs and cognition in healthy subjects has been studied; gene variants were found to be associated with differences in specific domains of cognitive function including IQ, executive functioning, and processing speed [220]. SNP2 rs1130233 was found to be associated with reduced expression of Akt1 in peripheral lymphocytes and brain grey matter in the same study, while another study which investigated obstetric complications in patients with schizophrenia, failed to find associations surviving correction for multiple testing [35]. This SNP was also associated with brain volume reductions in the caudate bilaterally and in the right prefrontal cortex [220]. It is possible that disruption of Akt regulation of GSK3 activity in the brain plays a role in dysregulation of brain function in schizophrenia, and that its restoration by antipsychotic medications may contribute to the clinical efficacy of these drugs. Consistent with these data, recent work has linked GSK3 to DISC1 [221, 222; see below].

In addition to rs1130233, another AKT1 SNP, rs2494732, was found to mediate long-lasting negative effects of psychosis on cognition (reduced performance on the continuous performance test) in people who had used cannabis, even if they had stopped using cannabis [223]. Both SNPs doubled the risk of being diagnosed with a psychotic disorder after cannabis use [224]. The authors speculated that cannabinoids may act down-stream the dopamine receptor to affect Akt-GSK3 interaction.

**Fig. (1):**
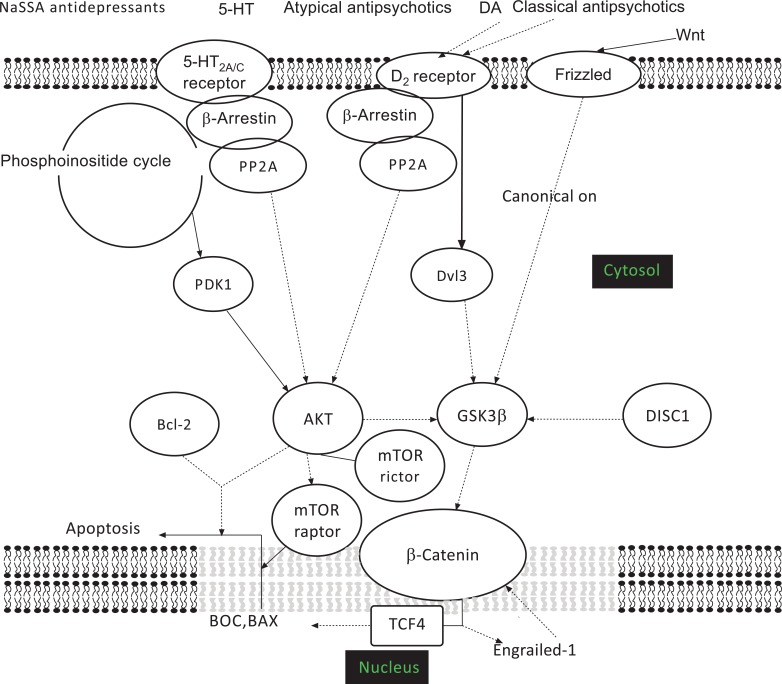
Simplified drawing of putative interactions between canonical Wnt and other pathways to illustrate their complex interplay. Dashed lines indicate inhibition, solid lines activation.

Gregorio *et al*. [194] investigated the possible association between structural brain alterations in patients with schizophrenia and 32 gene polymorphisms on 30 genes involved in neurogenesis and brain maturation (DNA extracted from peripheral blood mononuclear cells). Ventricular enlargement on 1.5 T magnetic resonance imaging (MRI) was associated with the reelin gene SNP rs362691 and impaired lateralisation of cortical gyrification was associated with the protocadherin 12 gene SNP rs164515; no significant associations were found for other structural abnormalities and gene polymorphisms, including the Wnt signalling-related *Wnt7A* SNP G/C (intron 2), *AKT1* SNP1 rs3730358, and *AKT1* SNP2 rs2498784. Taken together, these data indicate that schizophrenia may be associated with impaired neuronal migration and signalisation, and synaptic network specification. While the reelin pathway does not directly interact with Wnt signalling [225, 226], the cadherins display mutual interactions with the canonical Wnt pathway [227; Fig. **1**].

### 4.6. GSK3

Animal schizophrenia models are consistent with altered GSK activity. Significant reduction in GSK3β protein levels was found in the frontal cortex of the pre-puberty lesioned with excitotoxic hippocampal injury rats compared to post-pubertal rats and controls [228]. Another study showed increased inhibitory phosphorylation of GSK3β and β-catenin, but no significant changes in mRNA or protein levels in PFC and VTA of rats treated with MK-801, an NMDA glutamate-receptor antagonist which induces schizophrenia-like behaviours [229]. In the same study, increased APC mRNA and protein levels were found and matched enhanced β-catenin phosphorylation, suggesting that the latter is mediated through an APC effect on GSK3β. Doble *et al*. [230] produced mouse embryonic stem cells in which both alleles of GSK3α and GSK3-β were disrupted. Only in cells lacking three or all four alleles did a measurable increase in Wnt signaling occur; this indicates that a considerable proportion of GSK3 inhibition is required to increase the activity of the Wnt pathway.

A human study, which did not focus on brain alterations of GSK content or activity, also pointed to an alteration of GSK3 in schizophrenia. In fact, it found a reduction in both cellular activities and protein levels of kinase FA/GSK3α in the lymphocytes of patients with schizophrenia, as compared to healthy controls [231].

Post-mortem studies showed some inconsistencies; nevertheless they suggested GSK3 alterations in schizophrenia. A significant reduction in GSK3β [232], but not in β-catenin or Dvl-2 levels [233], was found post-mortem in the prefrontal cortex of 10 patients with schizophrenia, compared to 10 controls; however, this was not replicated when the same group of investigators extended their sample to 15 and compared it with 15 patients with bipolar disorder, 15 with unipolar depression and 15 with non-psychiatric conditions, with GSK-3β, β-catenin and Dvl-2 not differing among the groups [234]. In contrast, a subsequent post-mortem study failed to find changes in GSK3β expression in the occipital cortex of patients with schizophrenia, compared to bipolar and unipolar patients, and controls, but found decreased GSK activity in the frontal cortex of patients with schizophrenia [235]. Kozlovsky *et al*. [236] showed borderline reduction in dorsolateral prefrontal cortical mRNA levels of GSK3β, but not GSK3α, in patients with schizophrenia compared to other comparison groups [bipolar disorder, unipolar depression, and normal controls]. Another study failed to show significant differences in GSK3α and GSK3β mRNA levels in the frontal cortex of patients with schizophrenia, compared to healthy controls, but showed significantly lower hippocampal GSK3α and GSK3β mRNA levels in the patients with schizophrenia [237]. Based on the above findings, we may not infer as to the level of a basic alteration that may account for the multitude of the Wnt alterations found in post-mortem brain of patients with schizophrenia.

Dopamine receptor activation may have multiple effects on GSK3 function, which depend upon the receptor involved and whether they act through D_2_ receptor-mediated β-arrestin2/protein phosphatase 2A (PP2A) recruitment (increased Akt phosphorylation and release of GSK3 from Akt regulation) or through D_1_ and/or D_2_-mediated tyrosine kinase transactivation (enhanced Akt action and consequent GSK3 phosphorylation/inactivation) [238]. Antipsychotic drugs, who are believed to act through inhibiting mainly the D_2_ dopamine receptor, should be expected to inhibit the β-arrestin2/PP2A recruitment, thus enhancing the action of Akt and the phosphorylation of GSK3. However, the differential effects of these drugs on D_1_-D_2_ dopamine groups of receptors, as well as on other neurotransmitter receptors, account for a multitude of effects that have been described for this class of drugs.

Antipsychotic drugs like haloperidol, clozapine, risperidone and the experimental substituted benzoamide raclopride, a selective D_2_ blocker, induced an amphetamine-reversible increase in GSK3β levels in rat medial prefrontal cortex, striatum, ventral midbrain, and hippocampus without affecting its phosphorylation status [239, 240], as well as an increase in Dvl-3 levels in rat ventral forebrain and hippocampus [240]. However, another study found haloperidol and clozapine to differ in their ability to phosphorylate rat frontal cortical GSK3β, in that the effect of haloperidol was transient on Akt and poor on Dvl, while clozapine stably increased the amount of GSK3α/β phophorylation and Dvl [241]. Similarly, clozapine was found to activate Akt- and Dvl-mediated Ser^9 ^phosphorylation of GSK-3β in SH-SY5Y human neuroblastoma cells [242]. The atypical drug paliperidone (9-hydroxyrisperidone) was also found to act through the canonical Wnt pathway, inasmuch it inhibited the detrimental effect of the NMDA antagonist dizocilpine (MK-801) on Akt1 and GSK3β [243]. The differential effects of atypical *vs*. classical antipsychotics have been attributed to a preferential inhibition of the D_2_ receptor-mediated β-arrestin2/PP2A recruitment by the atypical antipsychotics, while classical neuroleptics like haloperidol would equipotently act through both the β-arrestin2/PP2A pathway and G protein signalling; this view was supported by the fact that GSK3β knock-out of D_2_ dopamine receptor-bearing mouse neurones mimicked the antipsychotic effect of aripiprazole, leaving unaffected the motor effect elicited by haloperidol (catalepsy) [244].

While the effect of antipsychotics on GSK-3β phosphorylation status is unclear, it is possible that some of the effects of antipsychotics are mediated through their actions on the Wnt pathway. Regretably, the post-mortem studies did not distinguish among the various antipsychotics received during their life by these patients, and this might have influenced the obtained results, as the effects of the various antipsychotics on the Wnt pathway vary [238, 245].

GSK-β function is under the control of DISC1 signalling, hence altered DISC1 could result in impaired Wnt signalling. DISC1 physically inhibits GSK3β activity, thus promoting subsequent phosphorylation and stabilization of β-catenin [220, 246, 247, Fig. **1**]. DISC1-mediated inhibition of GSK3β regulates neuronal precursor cell proliferation in both embryonic and adult mouse brains, further stressing the role of GSK3 and the Wnt pathway signalling in neurodevelop-ment [220]. While wildtype DISC1 effectively interferes with GSK3β signalling, some variants in many species are associated with the loss of GSK3β-regulating activity; for example, human lymphoblast cell lines endogenously expressing the R264Q DISC1 variant showed impairment in Wnt signalling and the S704C DISC1 variant was associated with impaired neuronal neocortical migration [221], pointing to a conjoint action of the DISC1 and Wnt signalling in neurodevelopment.

Genetic studies are not uniform in their conclusions about the involvement of GSK mutations or polymorphisms in schizophrenia. Several studies failed to find an association between mutations or polymorphisms of the *GSK3-β *gene and schizophrenia [248-253]. However, a case-control study examined GSK3β polymorphisms of the *GSK *gene SNPs rs7624540, rs4072520, and rs6779828 in patients with schizophrenia compared to healthy controls and showed a genotypic association between these SNPs and schizophrenia, suggesting that that this gene might be involved in the risk for schizophrenia [254]. The results of these studies were unfairly criticised [255], since the other studies claiming no association did not investigate the same SNPs as Souza *et al*. [254, 256]. Another study, analysing two common SNPs at position -1727 A/T and -50 C/T and a (CAA)(n) repeat polymorphism localized in intron1 of the *GSK3-β* gene in a group of patients with schizophrenia compared to healthy controls, showed that the allele, genotype, and haplotype distributions for the three polymorphisms investigated do not differ between patients and controls. However, (CAA)(3)/(CAA)(5) heterozygotes with the paranoid subtype of schizophrenia were more represented. These results support the reports that GSK3β appears to be involved in a subtype of schizophrenic patients, but not in schizophrenia in general [248]. Studying the effect of a GSK3-β SNP rs334558 on grey matter volumes of a group of patients affected by chronic schizophrenia, a recent study showed significantly higher brain volumes in an area encompassing posterior regions of right middle and superior temporal gyrus, within the boundaries of Brodmann area 21 in carriers of the rs334558 C allele, which is associated with reduced activity [168]. The temporal lobe is consistently associated with morphometric abnormalities in schizophrenia [168, 257, 258]. Summarising, it appears that the function of GSK3 is decreased in schizophrenia and its levels or phosphorylation increased by antipsychotic drugs, but neither the regions of decreased GSK activity are consistent (cortex and hippocampus are mostly affected, but the findings differ among studies), nor the effects of drugs are consistent, with benzoamides increasing levels, and butyrophenones and dibenzoazepines increasing phosphorylation, which ensues in decreased GSK3 activity and enhanced catenin formation. Genetic studies also show similar inconsistencies, across populations and across the SNPs reported to be associated. It is possible that *GSK3* activity alterations in schizophrenia appear to be so inconsistent because the important in the genetic expression of this disorder is gene interaction [259] or because *GSK3* is under some sort of epistatic control by other genes [260].

### 4.7. Adenomatous Polyposis Coli (APC)

A first post-mortem study [261] showed no significant differences in APC immunoreactivity (used as an oligodendrocyte marker) in any brain area among people with schizophrenia, bipolar disorder or healthy controls. A link with the glutamatergic hypothesis of schizophrenia [262] is provided by an animal study using the NMDA glutamate-receptor antagonist, MK-801 (dizocilpine), to induce schizophrenia-related behaviours in mice [229]. This study showed a significant increase in both APC mRNA and protein levels in the prefrontal cortex and the ventral tegmental area of mice displaying dizocilpine-induced schizophrenia-related behaviour, compared to controls. This may suggest that increased expression of APC is associated to attenuated Wnt pathway signalling in this animal model of schizophrenia. Backing the increased APC-induced Wnt down-regulation hypothesis, another study found an increased APC mRNA expression in leukocytes from patients with schizophrenia, which was independent from antipsychotic treatment [263]. This study also reported a significant association between APC mutations and schizophrenia using the transmission disequilibrium test (TDT) in Chinese trio families with schizophrenia; three SNPs at the exon region in the *APC* gene were found to be associated with schizophrenia, i.e., rs2229992, rs42427, and rs465899 [261]. Combining the above, it is likely that increased APC could impair Wnt signalling in schizophrenia and contribute to its pathology [263].

### 4.8. β-Catenin

A micro-array study of biopsies from olfactory epithelium from patients with schizophrenia, bipolar disorder, and healthy volunteers found a more than 50% reduction in the expression of pleiotrophin, a growth factor which enables β-catenin release from the plasma membrane, in schizophrenia [265]. The functional consequence of a strong reduction in pleiotrophin would be a reduction in β-catenin/Wnt signaling. B-cell CLL/lymphoma 9 protein is a protein that in humans is encoded by the *BCL9* gene [266]. BCL-9 contributes to efficient β-catenin-mediated transcription. In fact, it was shown that BCL9/Legless increased Wnt signal by promoting the transcriptional activity of β-catenin/Armadillo in normal and malignant cells [267].

Three studies examined copy number variations (CNVs) in schizophrenia cases and matched controls [268-270]. All these studies reported hemizygous *de novo* deletions at chromosome 1q21.1, an area comprising BCL-9. Although it is not yet known whether the haplo-insufficiency affects BCL-9 protein levels, it is conceivable that it leads or contributes to diminished Wnt signalling. However, a recent study showed various BCL-9 CNVs to be associated with schizophrenia in a Han Chinese population [271]. Mutations of an armadillo repeat gene (*ARVCF*), which is known to interact with β-catenin, was associated with the velo-cardio-facial syndrome [272], which is a disorder associated with 22q11.2 deletion and heart defects, facial dysmorphism and psychosis, which is a counterpart to the 22q11.2 deletion/DiGeorge syndrome, adding to the genetic complexity of 22q11.2 deletion.

Immunohistochemistry showed significantly reduced β- and γ-catenin post-mortem levels in the hippocampus of patients with schizophrenia compared to controls; such changes were not related to antipsychotic treatment [166]. However, another post-mortem study found no decrease in β-catenin levels in the prefrontal cortex of patients with schizophrenia, compared to healthy controls and to patients affected by other psychiatric disorders, such as bipolar disorder or unipolar depression [233].

Antipsychotic drugs like haloperidol, clozapine, raclopride, and risperidone induce increases in β-catenin levels in the rat medial prefrontal cortex, striatum [239], ventral midbrain, and hippocampus [240]; these effects were countered by amphetamine, a psychotogenic drug, indicating that D_2_ antagonism is important in promoting the effects of antipsychotic drugs on the Wnt pathway in these areas. 

### 4.9. TCF4

The *TCF4* gene, which maps in 18q21.2, encodes a basic helix-loop-helix (bHLH) transcription factor that interacts with other transcription factors to activate or repress gene expression. This protein belongs to the E-protein subfamily, which is known to be involved in neurodevelopment, but its function in the adult brain is still obscure [138, 273, 274]. TCF has Wnt/β-catenin-activated transcription factor properties [275]. Tcf4 transgenic mice with brain TCF4 overexpression display profound deficits in contextual and cued fear conditioning and sensorimotor gating, memory deficits and, importantly, deficits in prepulse inhibition; the latter is a neurophysiological correlate of schizophrenia and other psychiatric disorders [274]. On the other hand, *TCF4*-null/knock-out mice die in the first 24 h after birth [276-277]. In the human, *TCF4 *haploinsufficiency causes the Pitt–Hopkins syndrome, an autosomal dominant neuro-developmental disorder characterized by severe motor and mental retardation, microcephaly, epilepsy, and facial dysmorphisms [137, 278]. *TCF4*, like other MHC class I molecule alleles (*VRK2* and *ZNF804A*), were found in genome-wide association studies (GWAS) to be associated with schizophrenia in an Irish population [279]. The *TCF4* SNP rs2958182 was found to interact with diagnosis of schizophrenia on IQ and attention-related task performance in a Chinese Han population [280].

Investigating the impact of the rs9960767 SNP in intron 4 of the *TCF4 gene* on sensorimotor gating measured by prepulse inhibition (PPI) of the of the acoustic startle response in healthy humans and in patients with schizophrenia spectrum disorders, a statistically significant association between this SNP and schizophrenia was found [281]. Furthermore, in both samples, PPI was strongly decreased in carriers of the schizophrenia risk allele C of the *TCF4 *gene, whereas startle reactivity and habituation were unaffected by the TCF4 genotype [282]. TCF4 genotype sensorimotor gating modulation indicates a role for *TCF4 *gene variations in the development of early information-processing deficits in schizophrenia. If PPI is an appropriate endophenotype of schizophrenia, as proposed, a combination of these SNPs might also contribute to the risk of schizophrenia [281]. Patients with schizophrenia also displayed strong impairments of verbal declarative memory (VDM) function [283]. Carriers of the C allele of the rs9960767 polymorphism of the *TCF4* gene are less impaired in recognition, compared to those carrying the AA genotype. A trend toward higher scores in patients with the risk allele was found for delayed recall. The TCF4 genotype did not affect intelligence or total verbal learning score, but decreased VDM performance in patients with schizophrenia who carried the C allele of the rs9960767 polymorphism of the* TCF4* gene. However, the elevated risk for schizophrenia was not conferred by TCF4-mediated VDM impairment [284]. The rs9960767 polymorphism of the *TCF4* gene was shown in a GWAS to increase the risk of schizophrenia only slightly [285].

Seeking the mechanism at the *TCF* gene which could account for the association between the SNP rs9960767 and schizophrenia, a recent study examined *TCF4* for coding variants, and for cis-regulated variation in *TCF4* gene expression correlated with the associated SNP; this study used a SNaPshot assay to detect differential allelic expression measuring the relative expression levels of mRNA containing each exonic allele [286]. This study failed to identify any non-synonymous coding variants at the locus. Allele-specific expression analysis using human post-mortem brain samples obtained no evidence for cis-regulated mRNA expression related to genotype at this schizophrenia-associated SNP. This points to the association between schizophrenia and *TCF4* not being mediated by a relatively common non-synonymous variant, or by a variant that alters mRNA expression, as measured in adult human brain; however, it is still possible that the risk allele at this locus exerts effects on expression exclusively in a developmental context, in cell types or brain regions not adequately represented in Williams’ *et al*. analysis [286], or through post-transcriptional effects, for example, in the abundance of the protein or its sub-cellular distribution. However, the relationship between the rs9960767 SNP and schizophrenia is complex, since this SNP interacts with nicotine in modulating auditory sensory gating [287], and nicotine use is widespread among patients with schizophrenia [288]. A recent study identified a new schizophrenic risk factor at 18q21.2 rs4309482, which lies approximately equidistant from CCDC68 and TCF4, and could act through one of these genes or both [289]. Another mechanism, which may unify several genetic data obtained in schizophrenia, may be through the SNP rs1625579 of the *MIR137* gene, at 1p21.3 [290], which controls the production of microarray RNA miR-137, which in turn affects the function of other molecules, like CSMD1, C10orf26, CACNA1C, TCF4 [291], and ZNF804A [292], whose genes were found to be significantly associated with schizophrenia.

*TCF* gene variants which appear to be associated with schizophrenia are common variants, as opposed to the rare variants associated with neurodevelopmental disorders, and sometimes with the Pitt-Hopkins syndrome [293]. *TCF* appears not to mediate psychosis in general, as it is not involved in bipolar disorder, but rather psychotic traits specifically associated to schizophrenia [294, 295].

### 4.10. Dickkopf1

Dkk1 is encoded by the *DKK1* gene, which maps on chromosome 10 region q11.2, a region with linkage evidence for schizophrenia [178]. Dkk1 inhibits Wnt signaling by triggering LRP5/6 internalization through formation of a ternary complex with Kremen receptors [141, 296]. Kremen1, a high-affinity dickkopf homolog1, is *KREMEN1* gene-encoded transmembrane receptor mapping to chromosome 22q12.1, another region with linkage evidence for schizophrenia [297]. The involvement in schizophrenia of the *KREMEN1* locus appears to be mediated through the rs713526 single nucleotide polymorphism (SNP), which is located in the promoter region of *KREMEN1* and showed association with schizophrenia [298].

### 4.11. Dickkopf3

Gene expression analysis of the Wnt signalling antagonist Dickkopf-3 (Dkk3) has shown that its mRNA is down regulated in the temporal cortex of elderly people with schizophrenia [299]. The functional consequence regarding Wnt signalling is, in this particular case, unclear, since DKK3 is the only Dickkopf protein which apparently does not act as a Wnt antagonist [139].

### 4.12. Dickkopf4

An association study of 28 Wnt signalling genes found the polymorphic variation rs2073665 in the Dkk4 gene (*DKK4*) to be associated with schizophrenia [300]. Interestingly, the *DKK4* SNP, rs2073665 was found to be significantly associated with brain volume under both additive and dominant genetic models in 961 healthy Chinese individuals [301], thus strengthening the evidence for a neurodevelopmental hypothesis of schizophrenia.

### 4.13. Secreted Frizzled Related Protein1 (Sfrp1)

A possible connection of the *SFRP1* gene and neuropsychiatric disorders, including schizophrenia, has been advanced because of the location of the gene chromosome 8p, but to date no direct evidence for an association has been obtained [154].

Summarising genetic and post-mortem neuropathological findings of molecules or genes of Wnt-related signalling molecules in schizophrenia, we have to admit that genetic studies are much variable, despite the identification of many risk-conferring SNPs of Wnt-related genes, that replication studies all often do not confirm initial studies, that identified SNPs are not universal (i.e., they differ from population to population) and the added risk is generally low, while neuropathological studies are a little more consistent, with some studies identifying and other not confirming alterations of the same molecules, but at least showing involvement of the same molecules in the same direction, although the brain regions involved may differ. Neuropathological studies show increased Wnt1 in the hippocampus, decreased catenins and Akt1 in the hippocampus and decreased GSK function in cortex and hippocampus, although some of them did not find differences in GSK or GSK3 mRNA in cortex. Whether these differences may reflect the effect of long-term antipsychotic drug intake, is difficult to say based on reported data, as antipsychotics were usually reported as lifetime chlorpromazine equivalents with no differentiation among the various antipsychotic drugs. It should be emphasised that antipsychotics differ in the kinetics of Akt/GSK-3 phosphorylation, in the amount of post-drug exposure proteins, and in the proportion of pathway activation [245].

## 5. CONCLUSIONS

Schizophrenia is a multifactorial disease with onset in late adolescence-early adulthood. Among the genes putatively involved in its pathogenesis, many are those regulating the Wnt pathway and other pathways crossing their routes with the Wnt pathway. The strict connections of the Wnt signalling system with other intracellular pathways produces a sort of buffering that may dampen possible defects in few specific molecules, thereby putting the whole pathway lower in the pathophysiological hierarchy of schizophrenia. Although the entire picture is unclear, many Wnt-related molecules show abnormalities in schizophrenia, and may become a specific target for pharmacological intervention. The entire sytem, however, is so well buffered by other intracellular systems, that is not easy to anticipate the final effect of such a drug. It is much more reasonable to believe that a substance will facilitate the effect of currently available antipschotics, thus improving our ability to control schizophrenia. 

However, it is possible that studies of the involvement of Wnt pathways in schizophrenia and in the action of antipsychotic drugs will be of heuristic value in our attempt to “better characterize and subtype” this psychiatric entity. Current evidence supports that the concomitant expression of specific SNPs of various genes concur in determining specific behavioural traits, some of which are related to the expression of “schizophrenic” phenotypes. Some of these involve *ErbB4*, *NRG1*, and *KCNH2* isoforms; it should be recalled that the former two interact with the Wnt pathway in neural tissues. Future studies should focus on gene interaction in the expression of behaviours related to the heterogeneous entity we now call schizophrenia and on how antipsychotic treatment affects these interactions.

## Figures and Tables

**Table 1.** Summary of Post-mortem Studies of Wnt-related Molecules in Schizophrenia

Study	Investigated Association Between	Subjects	Results
J. Yang *et al*., 2003 [185]	rs2241802, rs2323019, rs352203 SNPs of *FZD3* gene and schizophrenia	246 Chinese Han family trios	Positive association between FZD3 locus and schizophrenia
Katsu *et al*., 2003 [187]	rs3757888, rs960914, rs2241802 SNPs of *FZD3* gene and schizophrenia	Japanese population; 200 unrelated schizophrenia patients and 218 healthy controls	Positive association between rs960914 and schizophrenia
Emamian *et al*., 2004 [203]	SNPs SNP5: rs2494732, SNP4: rs1130233A, SNP3: rs3730358, SNP2: rs1130214, SNP1: rs3803300 of *AKT1* gene and schizophrenia	210 proband-parent triads and 58 extended families, containing a total of 335 individuals affected with schizophrenia or schizoaffective disorder	One three-SNP haplotype (SNP2/3/4, TCG) is associated with schizophrenia
Ide *et al*., 2004 [189]	rs2241802, rs2323019, rs352203, rs352210, rs960914 SNPs of the *FZD3* gene and schizophrenia	Japanese population; 212 schizophrenia families[family samples]; 540 unrelated schizophrenia patients [case-control samples]	No association
Scassellati *et al*., 2004 [248]	Two common SNPs at position -1727 A/T and -50 C/T and a (CAA)(n) repeat polymorphism localized in intron 1 of the *GSK3β* gene	147 schizophrenia patients and 212 healthy controls	No association with schizophrenia but the (CAA)(3)/(CAA)(5) heterozygotes are more often represented in the subtype of paranoid schizophrenic patients
Wei and Hemmings, 2004 [190]	rs2241802, rs2323019, rs352203 SNPs of *FZD3* gene and schizophrenia	British population; 120 family trios (fathers, mothers and schizophrenic offspring)	No association
Zhang *et al*., 2004 [186]	2241802, rs2323019, rs352203, rs880481 SNPs of *FZD3* gene and schizophrenia	Chinese Han Population; 236 schizophrenia patients	Positive association between rs2323019 and rs880481 and schizophrenia
Cui *et al*., 2005 [263]	rs2229992, rs42427, rs465899 SNPs at the exon region of *APC* gene and schizophrenia; measurement of *APC* mRNA in leukocytes of patients with schizophrenia	Chinese population; six schizophrenic patients and six healthy controls. 59 patients with schizophrenia and schizophrenia-like disorders and 30 healthy controls. 163 parent- offspring trios	Presence of association; an increase mRNA is found in leukocytes.
Hashimoto *et al*., 2005 [191]	rs960914, rs2241802, rs2323019, rs352203 SNPs of *FZD3* gene and major psychosis	Japanese population; 427 patients with schizophrenia, 91 with bipolar disorder, 396 with major depression and 473 healthy controls	No association
Ikeda *et al*., 2005 [249]	rs4388007, rs2037547 SNPs of the *GSK3β* gene and schizophrenia, age-at-onset and subtypes of schizophrenia	Japanese population; 381 schizophrenic patients and 352 controls	No association
Lee *et al*., 2006 [250]	SNPs at position -1727 A/T, -50 C/T of *GSK3β* polymorphisms and schizophrenia	Korean population; 138 schizophrenia patients, 120 bipolar patients and 350 controls	No association
Szczepankiewicz *et al*., 2006 [251]	-50 T/C polymorphism of *GSK3β* gene and schizophrenia and bipolar disorder	402 schizophrenia patients, 416 bipolar patients and 408 controls	No association with schizophrenia
Pinheiro *et al*., 2007 [219]	rs2494732, rs2498799, rs3730358, rs1130241, rs3803300 *AKT1* SNPs and neurocognitive domains in schizophrenia	641 individuals with schizophrenia	No association
Xu *et al*., 2007 [214]	Five SNPs (rs3803300, rs1130214, rs3730358, rs2498799, rs2494732) of the *AKT1* gene and schizophrenia	Chinese population; 384 patients with schizophrenia *vs*. 384 healthy controls	G allele of rs3803300 SNP is associated; haplotype A-G-C-G-A constructed by 5 SNPs is associated
Kishimoto *et al*., 2008 [195]	rs3757888, rs960914, rs2241802, rs2323019, rs352203, rs880481 SNPs of the *FZD3* gene and methamphetamine psychosis	Japanese population; 188 patients with methamphetamine psychosis and 240 controls.	Haplotype frequency of G-A-T-G and A-G-C-A of rs2241802, rs2323019 and rs352203 SNP were significantly lower in patients with methamphetamine psychosis than in controls
J. Meng *et al*., 2008 [252]	rs334563, rs12630592, rs4688054, rs10934503, rs2319398 and rs4624596 SNPs of *GSK3β* gene and schizophrenia (case-control study)	Chinese population; 329 schizophrenia patients and 288 healthy controls	No association
Proitsi *et al*., 2008 [300]	50 SNPs in 28 Wnt signaling genes and schizophrenia	307 family-trios of Chinese origin; case-control samples of over 500 schizophrenia cases and 500 controls from Hong Kong	rs2073665 SNPs in *DKK4* gene is associated with schizophrenia
Souza *et al*., 2008 [254]	rs7624540, rs4072520, and rs6779828 SNPs of *GSK-3β* and schizophrenia	Caucasian population; 150 schizophrenia patients, 85 small nuclear families and 185 healthy controls	Positive association
Tan *et al*., 2008 [220]	rs2494732, rs1130233A, rs3730358, rs1130214, rs3803300, SNPs of *AKT1* gene and cognition in healthy subjects; effects of rs1130233 SNP of *AKT1* gene on AKT1 protein in peripheral lymphoblasts of healthy subjects; association between rs1130233 SNP of *AKT1* gene and prefrontal function and cortical DA function using fMRI in healthy subjects; association between rs1130233 SNP of *AKT1* gene and brain structure using MRI Family-based and a non-independent case-control association study: association between rs2494732, rs1130233A, rs3730358, rs1130214, rs3803300, SNPs of *AKT1* gene and schizophrenia	European population 319 individuals for cognitive tests 32 healthy controls for AKT1 protein expression in lymphoblasts 46 and 68 healthy subjects for fMRI 171 individuals for structural brain MRI 358 unrelated probands and 370 unrelated healthy controls for family-based and a non-independent case-control association study	Positive association is found between rs 1130233 SNP and brain volume reductions in the caudate bilaterally and right prefrontal cortex in patients with schizophrenia rs1130233 is associated with reduced performance on cognitive functions, with reduced AKT1 protein levels, with increased prefrontal activation (inefficient activation) rs1130233 is associated with relatively reduced gray-matter volumes in the bilateral caudate and right prefrontal cortex rs1130233 is associated with increased risk for schizophrenia in the case control dataset but not in the non-independent family-based association study
Thiselton *et al*., 2008 [205]	rs1130214, rs2494732, rs2498799, rs3730358 rs2494746, rs3803304, rs2498802, rs2498804 SNPs of the *AKT1* gene and schizophrenia	Irish population; 265 high-density schizophrenia families with 1408 individuals available for genotyping.	No association
Gregorio *et al*., 2009 [194]	G/C (intron 2) of *Wnt7A* gene, SNP1 rs3730358, SNP2 rs2498784, of *AKT1* gene and MRI morphological alterations observed in 25 patients with schizophrenia; associations between impaired left/right gyrification index and protocadherin 12 gene SNP rs164515, and between ventricular enlargement and reelin gene SNP rs362691	Twenty-five schizophrenic patients	No association
Stefansson *et al*., 2009 [281]	rs9960767 SNP of *TCF4* gene and sensorimotor gating in schizophrenia	European population; 2663 patients with schizophrenia and 13498 controls	Positive association between rs9960767 SNP and schizophrenia
Aleksic *et al*., 2010 [298]	rs713526 SNP in the promoter region of *KREMEN1* and schizophrenia	Japanese population; 1624 patients with schizophrenia and 1621 healthy volunteers	Presence of association
Benedetti *et al*., 2010 [168]	rs334558 SNP of *GSK3β* gene and grey matter volumes (voxel-based morphometry) in schizophrenic patients	57 schizophrenia patients	Carriers of C allele variant showed higher brain volumes in an area comprising posterior regions of right middle and superior temporal gyri
H.J. Kim *et al*., 2010 [72]	rs2024233, rs1051751, rs733154, rs3779548, rs17132543, rs6948009, rs4730775, rs39315 SNPs of the *Wnt2* gene and schizophrenia	Korean population; 288 patients with schizophrenia and 305 healthy controls	Weak positive association between rs4730775 and schizophrenia only, none for other SNPs
C. Kang *et al*., 2011 [193]	*FZD3* SNPs rs2241802, rs2323019, rs352203, rs880481, and rs3757888 and schizophrenia	81 Va Chinese patients with schizophrenia *vs*. 210 healthy controls	Haplotype analyses showed significant differences between patients and controls at rs2241802 only, after Bonferroni correction, and differences at at rs2241802-rs2323019-rs352203 haplotype
Lennertz *et al*., 2011 [284, 285]	C allele of the rs9960767 polymorphism of the *TCF4* gene and verbal declarative memory (VDM) functions in schizophrenia	401 patients with schizophrenia	Carriers of the C allele were less impaired in recognition compared to those carrying the AA genotype
J. Li *et al*., 2011 [271]	*BCL-9* SNPs rs9326555, rs10494251, rs1240083, rs672607, rs688325, and rs3766512 and schizophrenia, bipolar disorder or major depressive disorder	4187 Chinese Han patients with schizophrenia *vs*. 5772 healthy controls *vs*. 1135 patients with bipolar disorder *vs*. 1135 patients with major depressive disorder	Association between SNPs rs9326555, rs10494251, rs1240083, rs672607 (strongest), rs688325, and rs3766512 and schizophrenia, rs672607 and bipolar disorder, and rs672607, rs1541187, rs688325, rs10494251, rs946903 and major depressive disorder

C=Controls; ELISA=Enzyme-linked immuno sorbent assay.

## ABBREVIATIONS

ACP1: = acid phosphatase 1
Akt: = Protein kinase B/Non-specific serine/threonine protein kinase family
APC: = Adenomatous poliposys coli
BP: = Bipolar disorder
BMPs: = Bone Morphogenetic Proteins
Ca^2+^: = Calcium ion
Cdc42: = Cell division control protein 42
CNV: = Copy number variation
CUS: = Chronic unpredictable stress
DAG: = Diacylglycerol
Dvl: = Dishevelled
EPM: = Elevated plus maze
FGF: = Fibroblast growth factor
Fzd: = FZD, Frizzled
GM: = Grey matter
GSK3(α/β): = Glycogen synthase kinase-3(alpha, beta))
HDAC: = Histone deacetylase
InsP3: = Inositol-1,4,5-trisphosphate
LRP5/6: = Low Density Lipoprotein receptor-related protein 5 and -6
LTP: = Long-term potentiation
MAPK: = Mitogen-activated protein kinase
Mg^2+^: = magnesium ions
MDD: = Major Depressive Disorder
NLK: = Nemo-like kinase
PPARD: = Peroxisome proliferators-activated receptor type-δ
PCP: = Planar Cell Polarity
PI3K: = Phosphatidylinositol-3-kinase
PKC: = Protein kinase C
ROCK: = Rho-associated kinase
SHH: = Sonic Hedgehog
SNP: = Single Nucleotide Polymorphism
TAK1: = Transforming growth factor-β-activated protein kinase
TCF/LEF: = T-cell factor/lymphoid enhancer factor family of transcription factors
Wnt: = Wingless
